# Detection of the Dimorphic Phases of* Mucor circinelloides* in Blood Cultures from an Immunosuppressed Female

**DOI:** 10.1155/2016/3720549

**Published:** 2016-09-29

**Authors:** Miguel A. Arroyo, Bryan H. Schmitt, Thomas E. Davis, Ryan F. Relich

**Affiliations:** ^1^Department of Pathology and Laboratory Medicine, Indiana University School of Medicine, Indianapolis, IN 46202, USA; ^2^U.S. Army Medical Department Center and School, Fort Sam Houston, TX 78234, USA

## Abstract

*Mucormycosis* fungemia is rarely documented since blood cultures are nearly always negative. We describe a case of* Mucor circinelloides* fungemia in a patient with a history of a sinus infection, sarcoidosis, and IgG deficiency. The identity of the isolate was supported by its microscopic morphology and its ability to convert into yeast forms under anaerobic conditions. The early detection, initiation of liposomal amphotericin B treatment, and reversal of underlying predisposing risk factors resulted in a good outcome.

## 1. Introduction

Mucormycoses are infectious diseases caused by filamentous fungi classified among the order Mucorales [1]. Infection occurs when a susceptible (e.g., immunocompromised) human is exposed to spores through inhalation, ingestion, or traumatic implantation. The resulting disease processes are characterized by an often rapid clinical progression and a high mortality rate [1, 2]. Mucorales rarely cause infection in immunocompetent persons but can cause fatal infection in immunocompromised patients. Risk factors include hematologic malignancies, bone marrow or solid organ transplantation, neutropenia, diabetes mellitus, corticosteroids, iron chelation therapy, broad spectrum antibiotics, antifungal prophylaxis, prolonged voriconazole use, cutaneous breakdown (from trauma, surgical wounds, needle-sticks, or burns), hyperalimentation, and severe malnutrition [1, 2]. Features of these organisms that contribute to their virulence include their rapid growth rate and their ability to invade blood vessels [1–3]. The latter characteristic permits their spread throughout the body, enabling access to numerous tissues and organs that may ultimately become infected [2]. However, documented mucormycosis fungemia is very rare, and blood cultures are usually negative. Demonstration of invasive disease by these organisms generally requires the identification of fungal elements directly in clinical specimens via histopathological examination or growth of these organisms from more than one specimen obtained from a normally sterile site [3].

Several* Mucor* spp., but not all, are known to be dimorphic. This distinct group of zygomycetes exhibits hyphal growth in aerobic conditions and multibudded yeast growth under anaerobic or high-CO_2_ conditions [4].* M. circinelloides* is one of these species, along with* M. racemosus*,* M. rouxii*,* M. genevensis*,* M. bacilliformis*,* M. subtilissimus*, and* M. amphibiorum*.* M. circinelloides* infections are well documented in the literature and have been associated with cutaneous, rhinocerebral, and pulmonary infections [5–9]. To our knowledge, only two cases of fungemia caused by this organism have been reported [10, 11]. In this report we describe a case of* M. circinelloides* fungemia in a female with a prior history of chronic sinus infection, sarcoidosis, and IgG deficiency.

## 2. Case Report

**Figure 1:**
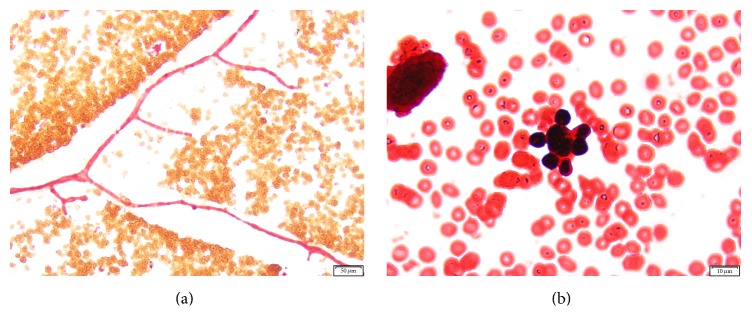
Gram stain of* M. circinelloides* dimorphic phases from a positive blood culture. (a) Pauciseptate wide hyphae with right-angle branching (100x magnification). (b) Circumferentially budding yeast resembling the yeast phase of* Paracoccidioides brasiliensis* (500x magnification).

A 38-year-old female presented to the Emergency Department of a hospital in rural Indiana with shortness of breath and cough, sinus congestion, fevers as high as 39.4°C, nasal drainage, sore throat, and yellow-green sputum production. The patient's medical history included sarcoidosis, IgG deficiency, and a sinus infection for which she completed treatment with cefdinir 1 week before admission. The patient stated improvement while on antibiotics but symptoms worsened after therapy completion. Upon physical examination, the patient had a temperature of 37.5°C and exhibited moderate respiratory distress with no wheezing, rales, or rhonchi. She was alert, awake, and oriented with no ear, nose, throat, cardiovascular, abdominal, back, extremities, skin, neurological, or psychiatric abnormalities. An X-ray of her chest was unremarkable. Peripheral blood specimens obtained at the time of initial presentation demonstrated leukocytosis, hyperglycemia and low potassium, CO_2_, total protein, and magnesium (Table 1). The aerobic blood culture bottles (BD BACTEC*™* Plus Aerobic/F) from two sets of blood cultures collected at the time of admission turned positive within 24 hours of incubation in a continuous-monitory blood culture system (BACTEC*™* 9240, BD Diagnostics, Sparks, MD). Gram stains of the broth revealed pleomorphic yeast forms and wide, pauciseptate, hyphae with right-angle branching (Figure 1(a)). Yeast forms with single, bipolar, and circumferential buds were observed. Several of the yeast cells observed resembled the yeast phase of* Paracoccidioides brasiliensis* (Figure 1(b)). The Gram stain findings were reported as “fungal elements present” and were immediately phoned to the clinical care team.

**Figure 2:**
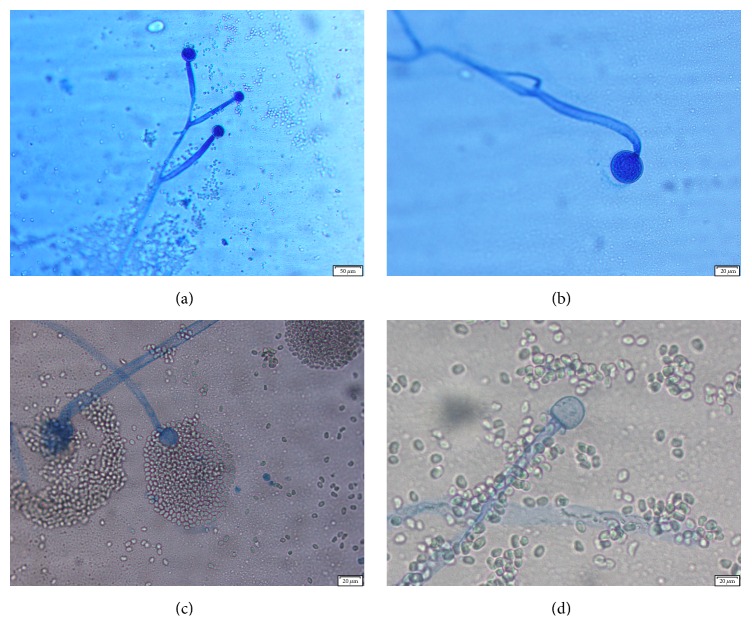
Microscopic examination of* Mucor circinelloides* on PDA by “tape prep” using lactophenol cotton blue stain. (a) Sympodially branched sporangiophores (100x magnification); (b) circinate sporangiophores (200x magnification); (c) deliquescent sporangia (100x magnification); (d) columella with collarette (200x magnification).

**Figure 3:**
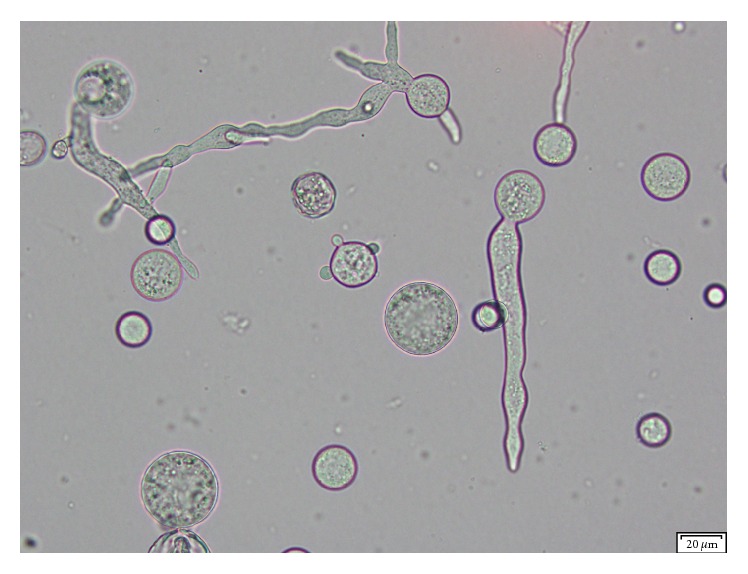
*Mucor circinelloides* yeast forms after cultivation of the isolate on Sabouraud dextrose agar incubated for 48 hrs under anaerobic conditions showing hyphae with single and multipolar buds (unstained, 200x)

The following day, mold growth was observed on the aerobic sheep blood agar and chocolate agar plates incubated at 35°C in 5% CO_2_ and on potato dextrose and Sabouraud agars incubated at 30°C. Fast-growing, floccose colonies were initially pale gray to yellow, turning brown over time when incubated at 35°C. Microscopic examination of the mold colony by lactophenol cotton blue staining revealed broad, ribbon-like pauciseptate wide hyphae with right-angle branching. Sympodially branched and circinate sporangiophores were observed (Figures 2(a) and 2(b)). Globose sporangia measured within 25–80 *μ*m and contained oval shaped sporangiospores (Figure 2(c)). Columellae were spherical, measuring up to 50 *μ*m in diameter; collarettes were present (Figure 2(d)). Rhizoids and stolons were absent. The identity of the isolate as* M. circinelloides* was supported by its microscopic morphology, its ability to convert into yeast forms under anaerobic conditions, and its inability to grow at 42°C (Figure 3). Conclusive identification based on molecular and phenotypic methods was above the scope of this study. Following reporting of these findings, the patient was started on liposomal amphotericin B 500 mg IVPB every 24 hours; subsequent blood and sinus cultures were negative. In addition, sinus biopsy results and computerized tomography (CT scan) of the chest and sinuses did not show any clear evidence of fungal involvement. As a consequence, antifungal treatment was discontinued after 6 days and the patient was discharged home without further complications. The patient had no signs of* Mucor* infection 7 months after discharge.

## 3. Discussion

The saprophytic nature of the Mucorales makes the diagnosis of mucormycosis a challenging task when these organisms are isolated from nonsterile sites [1]. Because they are ubiquitous, the presence of these organisms can sometimes be dismissed, as, in many cases, they likely represent contamination of the clinical specimen or culture media. Therefore, it is essential that the physician and laboratory personnel work together to determine the clinical significance of the laboratory results. In most instances, the diagnosis of invasive mucormycosis is usually made when these organisms are identified directly from clinical specimens or isolated in culture from more than one specimen obtained from a normally sterile site. In the case presented, the diagnosis of mucormycosis was made by the isolation of* Mucor circinelloides* from two blood culture sets that were collected from two different sites. This finding was considered clinically significant since it represented multiple specimens obtained from a normally sterile site.

In this case, the source of infection is unknown, but we hypothesized that is likely related to her sinusitis. The successful treatment and optimal outcome were most likely attributed to the early detection and isolation of the organism from blood cultures, which prompted early implementation of antifungal therapy. In addition, the correction of her hypokalemia, hypomagnesemia, and hyperglycemia contributed to her recovery, as reversal of underlying physiological dyshomeostatic conditions has been reported to correlate with good patient outcomes [12]. Surgical debridement was not necessary since follow-up tests did not reveal foci of fungal infection. Based on the patient outcome, we can also hypothesize that this case might have represented a transient fungemia or that the causative organism is of low virulence [13].

To our knowledge, only two cases of fungemia associated with* M. circinelloides* have been previously described. The first reported cases occurred in a 48-year-old male with a history of short-gut syndrome secondary to multiple abdominal surgeries [10]. This patient had a central venous catheter (CVC) that was required for chronic total parenteral nutrition. Blood cultures obtained from the CVC on consecutive days grew both* Candida albicans* and* M. circinelloides*. Treatment with liposomal amphotericin B was started, and the CVC was removed. Similar to our case, the authors stated that early diagnosis of mucormycosis coupled with rapid therapeutic intervention led to a successful outcome. The second reported case was that of an 83-year-old diabetic woman who developed an acute left frontoparietal infarct while hospitalized in a neurological intensive care unit [11]. The initial isolation of a mold from a blood culture was considered to be a contaminant, but during the following days, the patient developed an erythematous and edematous lesion on her right hand. A skin biopsy culture grew a mold identical to that in the blood culture; both isolates were later identified as* M. circinelloides* by molecular methods. Despite treatment, the patient's clinical condition worsened and she later died.

## 4. Conclusion

This case represents the third documented case of fungemia cause by* M. circinelloides*. The most likely underlying factors were probably related to her chronic sinus infection, IgG deficiency, and chronic steroid use. The detection of this mold and reversal of the predisposing underlying conditions were vital in the successful treatment.

## Figures and Tables

**Table 1:** Clinical laboratory test results from blood specimens obtained at the time of patient presentation to the ED.

Test	Patient result	Reference range
WBC	13.7k/cumm	3.6–5.17k/cumm
Hgb	11.5 GM/dL	12.0–16.0 GM/dL
MCV	79 fL	81–99 fL
MCH	24.9 pg	27–34 pg
MCHC	31.5 GM/dL	32.0–36.0 GM/dL
RDW	15.8%	11.5–14.5%
Absolute neutrophil	8.2k/cumm	1.7–7.6k/cumm
Absolute lymphocyte	4.0k/cumm	1.0–3.2k/cumm
Absolute eosinophil	0.4k/cumm	0.0–0.3k/cumm
Potassium SerPl QN	2.8 mmol/L	3.5–5.5 mmol/L
Carbon dioxide SerPl QN	21 mmol/L	22–29 mmol/L
Glucose SerPl QN	138 mg/dL	70–99 mg/dL
Magnesium SerPl QN	1.3 mg/dL	1.6–2.9 mg/dL
AST SerPl QN	8 units/L	13–39 units/L
Total protein SerPl QN	6.0 GM/dL	6.4–8.0 GM/dL
